# Vitamin E and Its Molecular Effects in Experimental Models of Neurodegenerative Diseases

**DOI:** 10.3390/ijms241311191

**Published:** 2023-07-07

**Authors:** Bianca Caroline da Cunha Germano, Lara Cristina Carlos de Morais, Francisca Idalina Neta, Amélia Carolina Lopes Fernandes, Francisco Irochima Pinheiro, Amália Cinthia Meneses do Rego, Irami Araújo Filho, Eduardo Pereira de Azevedo, José Rodolfo Lopes de Paiva Cavalcanti, Fausto Pierdona Guzen, Ricardo Ney Cobucci

**Affiliations:** 1 Postgraduate Program in Science Applied to Women’s Health, Federal University of Rio Grande do Norte (UFRN), Natal 59072-970, Brazil; bianca@ufrn.br (B.C.d.C.G.); ricardo.cobucci.737@ufrn.edu.br (R.N.C.); 2 Postgratuate Program in Health and Society, Department of Biomedical Sciences, Faculty of Health Sciences, State University of Rio Grande do Norte (UERN), Mossoró 59607-360, Brazil; laracarlos@alu.uern.com.br (L.C.C.d.M.); rodolfolopes@uern.br (J.R.L.d.P.C.); 3 Laboratory of Experimental Neurology, Department of Biomedical Sciences, Faculty of Health Sciences, State University of Rio Grande do Norte (UERN), Mossoró 59607-360, Brazil; franciscaidalina@alu.uern.br (F.I.N.); ameliacarolina@alu.uern.br (A.C.L.F.); 4 Postgraduate Program in Physiological Sciences, Department of Biomedical Sciences, Faculty of Health Sciences, State University of Rio Grande do Norte (UERN), Mossoró 59607-360, Brazil; 5 Postgraduate Program in Biotechnology, Health School, Potiguar University (UnP), Natal 59056-000, Brazil; irochima@gmail.com (F.I.P.); irami.filho@gmail.com (I.A.F.); azevedoep@hotmail.com (E.P.d.A.); 6 Medical School, Health School, Potiguar University (UnP), Natal 59056-000, Brazil; 7 Postgraduate Program in Health Sciences, Federal University of Rio Grande do Norte (UFRN), Natal 59078-900, Brazil

**Keywords:** experimental models of neurological diseases, vitamin E, alpha tocopherol, experimental models

## Abstract

With the advancement of in vivo studies and clinical trials, the pathogenesis of neurodegenerative diseases has been better understood. However, gaps still need to be better elucidated, which justifies the publication of reviews that explore the mechanisms related to the development of these diseases. Studies show that vitamin E supplementation can protect neurons from the damage caused by oxidative stress, with a positive impact on the prevention and progression of neurodegenerative diseases. Thus, this review aims to summarize the scientific evidence of the effects of vitamin E supplementation on neuroprotection and on neurodegeneration markers in experimental models. A search for studies published between 2000 and 2023 was carried out in the PubMed, Web of Science, Virtual Health Library (BVS), and Embase databases, in which the effects of vitamin E in experimental models of neurodegeneration were investigated. A total of 5669 potentially eligible studies were identified. After excluding the duplicates, 5373 remained, of which 5253 were excluded after checking the titles, 90 articles after reading the abstracts, and 11 after fully reviewing the manuscripts, leaving 19 publications to be included in this review. Experiments with in vivo models of neurodegenerative diseases demonstrated that vitamin E supplementation significantly improved memory, cognition, learning, motor function, and brain markers associated with neuroregeneration and neuroprotection. Vitamin E supplementation reduced beta-amyloid (Aβ) deposition and toxicity in experimental models of Alzheimer’s disease. In addition, it decreased tau-protein hyperphosphorylation and increased superoxide dismutase and brain-derived neurotrophic factor (BDNF) levels in rodents, which seems to indicate the potential use of vitamin E in preventing and delaying the progress of degenerative lesions in the central nervous system.

## 1. Introduction

Over the past few years, as a result of the steady rise in life expectancy, a significant increase in the prevalence of neurodegenerative diseases (NDD) has been evident, which has resulted in increased health risks and more demanding research into new and more efficient therapies [1]. In addition, the great majority of NDD are debilitating and untreatable, resulting in suffering for the affected patients and their families [2,3,4].

The disorders start by affecting neurons in a gradual process, resulting in degeneration and/or death of some neurons, which might lead to impaired physical movements and cognitive function [5]. In fact, some neurodegenerative diseases, such as Alzheimer’s disease (AD), Parkinson’s disease (PD), and dementia with Lewy bodies, have some common clinical manifestations such as abnormal protein deposition, inflammation, mitochondrial deficits, intracellular Ca^2+^ overload, abnormal cellular transport, uncontrolled generation of reactive oxygen species (ROS), and excitotoxicity. Thus, the existence of convergent neurodegeneration pathways in such diseases becomes clear [6]. However, there are still no therapeutic strategies that can prevent, reduce, or stop the progression of these diseases, even though some drugs are able to treat only the symptoms [7].

Some natural products exhibit a variety of neuroprotective activities, which include targeting mitochondrial dysfunction, excitotoxicity, inflammation, apoptosis, oxidative stress, and protein folding [8,9,10,11,12]. One of these products from natural sources with reported neuroprotective activity is vitamin E (VE), which is formed by different fat-soluble compounds found in plants and is divided into tocopherols and tocotrienols. Each type is formed by four homologs according to the number and location of the methyl groups, being classified as α-, β-, γ-, and δ-tocopherol and as α-, β-, γ-, and δ-tocotrienol [13].

The effects of α-tocopherol (α-Toc) on the central nervous system (CNS) have been reported for over 50 years [14]. A relationship between brain health and α-tocopherol levels has been hypothesized, especially in diseases associated with oxidative stress such as ataxias, AD, and PD [15]. Therefore, VE deficiency might lead to progressive neurological disorders such as spinocerebellar ataxia as a result of the death of peripheral nerves. Thus, long-term α-Toc supplementation may prevent the progression of nervous system degeneration caused by VE deficiency [16,17].

In this perspective, both tocopherol transfer protein (TTP) and vitamin E have important functions in the CNS, as they are necessary for embryonic development, neurogenesis, neuroprotection, and cognition [18]. Furthermore, it acts as a peroxyl-radical-scavenging antioxidant that inhibits free radical-mediated lipid peroxidation [19]. Moreover, studies indicate that α-Toc reduces lipid peroxidation caused by lipopolysaccharide (LPS), microglia, and interleukin-6 (IL-6) [20,21], in addition to having a positive effect on neuroplasticity [22]. In fact, αToc is able to break the oxidant chain found in lipoproteins and cellular compartments such as cell membranes, preventing lipid peroxidation and thus preserving membrane integrity [23,24].

Ambrogini et al. reported that α-tocopherol can protect against kainate-induced cell death in the hippocampus, thereby preserving its synaptic plasticity and function [25]. Moreover, other studies have evidenced the antioxidant activity of VE and αToc in the CNS with significant improvements in memory and motor function, as well as a marked increase in the levels of neuroprotective, neuroregenerative, and anti-inflammatory components [26,27]. However, there are a very limited number of reviews about the effects of VE supplementation on animal models of neurodegenerative diseases.

Considering that the studies published so far show positive effects of VE and αToc on the CNS, this narrative review aims to analyze the scientific evidence of the effects of VE and αToc supplementation in animal models of neurodegenerative diseases.

## 2. Methodology

A search for studies published between January 2000 and April 2023 was carried out in Medline databases through PubMed, Web of Science, Virtual Health Library (BVS), and Embase, in which the effects of vitamin E in experimental models of neurodegeneration were investigated. The search strategy consisted of a combination of Medical Subject Headings (MeSH) terms “neurodegenerative diseases” and “alpha-tocopherol” (Medline and Web of Science); “degenerative disease” and “alpha-tocopherol” (Embase); and “chronic disease” and “alpha-tocopherol” (BVS). The search strategy performed in PubMed can be verified in the Supplementary Material Table S1.

Studies involving the effects of VE on memory, cognition, learning, motor coordination, neuroprotection, neuroregeneration, inflammatory markers, and biomarkers of oxidative stress in experimental models of neurodegeneration were included in this review. Studies that met one of the following criteria were excluded: (1) Review studies; (2) Non-peer-reviewed studies (such as guidelines and preprints); (3) studies performed with vitamin E associated with other herbal supplements, drugs, or therapies; (4) double publication: if the article appeared more than once in one of the databases, only the original manuscript was included; (5) experimental models other than small rodents (rats and mice) or cell culture; (6) studies carried out with extracts of more than one oil or with the use of parts of the plant that are not the seed, such as the stem, leaf, fruit, root, and flower.

The results of the eligible studies are described herein as a narrative synthesis that summarizes the characteristics of the study, the studied population (animals), as well as the type of VE and αToc supplementation used.

## 3. Results

The search strategy used in this study resulted in 5669 potentially eligible articles. After excluding the duplicates, 5373 articles remained, of which 5253 were excluded after checking the titles, 90 after reading the abstracts, and 11 after the complete review of the manuscripts, leaving a total of 19 publications to be included in this review.

The included studies are presented in Table 1 with the following information: author, year of publication, species, control group, induction method, experimental model of neurodegeneration, type of VE supplementation and administration method, supplementation time and data collection, the dose used, and the main results.

### 3.1. Neuroprotective Mechanisms of Vitamin E in Neurodegenerative Diseases

#### 3.1.1. Memory and Learning

**Figure 1:**
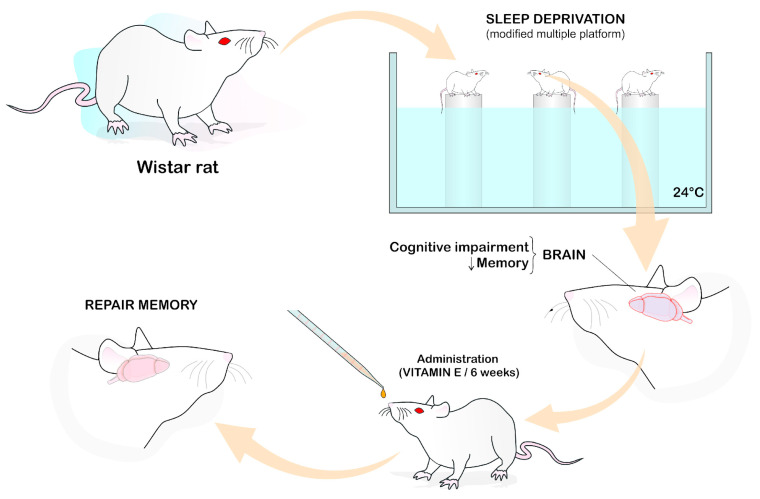
Vitamin E and memory. Illustration demonstrates that sleep deprivation impairs cognition/memory. The role of vitamin E in memory repair after its administration for 6 weeks in Wistar rats with sleep deprivation-induced memory impairment using the modified multiple platform method. (Illustration performed by Dr. Francisco Irochima).

The study by Alzoubi [35] was carried out using Wistar rats as the animal model, in which they were deprived of sleep followed by oral administration of VE (100 mg/kg) for six weeks. Such a procedure was able to prevent memory impairment (Figure 1).

Desrumaux et al. used PLTO-KO mice that were deficient in plasma phospholipid transfer and were induced to develop memory deficit and Alzheimer’s disease (AD) through β 25–35 administration. The animals were supplemented with vitamin E (800 mg/kg orally) for 10 days, whose effect on short-term memory impairment was considered satisfactory [36].

Wang et al. conducted their studies using APPswe/PS1dE9 transgenic mice. After developing AD-triggering Aβ deposits, the animals received αToc, 100 mg/kg, orally for four weeks. The authors concluded that supplementation with αToc had positive effects on memory preservation [39].

Nesari et al. supplemented αToc (60 and 200 mg/kg) for five days after causing proteasome inhibition and memory deficit in Wister rats through lactacystin induction. The authors reported benefits only in those rats that received the highest doses of αToc [43].

Finally, Shadini et al. induced AD in Wistar rats through β 25–35 administration. VE supplementation (200 mg/kg orally) for 10 days resulted in the passive avoidance of memory impairment [44].

In addition, other studies investigated the effectiveness of VE and αToc on learning. The experiment conducted by Conte et al. with AD-induced Tg 2576 mice investigated the administration of 2 IU/g of VE for four weeks before repetitive concussive brain injury (RCBI), followed by another eight weeks of vitamin supplementation after the onset of AD. The authors reported a potential effect of VE supplementation on alleviating the learning deficit [29].

Likewise, Annaházi et al. studied the effect of α-Toc administration in Wistar rats with chronic cerebral hypoperfusion generated by brain injury in the bilateral common carotid arteries. The α-Toc administration (100 mg/kg) for five days before the injury and the same dose for another five days after the injury was able to improve the learning process [31].

#### 3.1.2. Cognitive

Concerning the effects of VE on cognitive functions, a study investigated the administration of α-Toc (100 mg/kg) for 21 days to Wistar rats subjected to streptozotocin (ST’Z) injury for AD and cognitive deficit induction. Such a protocol was able to successfully prevent cognitive impairment by protecting the animals’ brains against oxidative stress and eliminating nitrosative stress and acetylcholinesterase activity [33].

Ishihara et al. obtained similar results, but with a dose of α-TOH of 1.342 mg/kg for six months in 3 tg-AD mice with AD and cognition impairment triggered by Aβ accumulation in the brain. This dose of α-TOH was able to reduce oxidative stress by attenuating the expression of the brain-derived neurotrophic factor [37].

Wang et al. reported improvement in cognitive dysfunction related to spatial memory due to a protective effect that resulted in a reduction in lipid peroxidation and a decrease in the release of inflammatory mediators (IL-1 and IL-6) through the oral administration of α-Toc (100 mg/kg for four weeks) in transgenic APPswe/PS1dE9 mice previously induced to develop AD [39].

Liu et al. used C57BL/6J transgenic mice in which PM 2.5 was administered to induce cognitive deficit. The administration of VE (60 mg/kg/day for seven days) resulted in improved cognitive function, which was correlated with the ability to memorize and learn on the Morris water maze test (MWM), suggesting that it was associated with reduced oxidative stress [40].

Finally, Rana et al. used α-Toc, 5–10 mg/kg over 28 days after induction of traumatic brain injury (TBI) in Wistar rats, resulting in reduced cognitive impairment. The use of such a protocol of α-Toc supplementation reduced neuroinflammatory markers, restored neurotransmitter levels, and restored oxidative stress balance [42].

#### 3.1.3. Motor Coordination

The studies included in this section investigated whether VE and α-Toc supplementation generated a positive impact in animal models with motor and behavioral impairments, even though they used different experimental models.

In one study, Tg 2576 mice were induced to develop AD through repetitive concussive brain injury (RCBI) in the left parietal-temporal region. Briefly, 2 IU/g of VE was administered for four weeks before and eight weeks after the lesion, and an improvement in behavior caused by RCBI was observed with cerebral lipid peroxidation secondary to brain trauma [29].

In another study, Sprague-Dawley rats were used, and they were fed for five weeks with α-Toc (20 mg/kg/day, orally) after being induced to develop orofacial dyskinesia and vacuum chewing movements (MVC) by haloperidol (HAL). It was observed a reduction in the stereotyped behavior of chewing movements after α-Toc administration [38].

Finally, Wistar rats were induced to exhibit motor impairment by a TBI lesion, whose locomotor deficit was attenuated when supplemented with 5–10 mg/kg of α-Toc for 28 days [42].

#### 3.1.4. Oxidative Stress and Neurodegenerative Diseases

VE supplementation can cause changes in the CNS and positively impact oxidative stress. In the study conducted by Sung et al., AD-induced Tg 2576 mice were supplemented with vitamin E (2 IU/g) for 8 months. The authors found a reduction in lipid peroxidation as well as a decrease in the levels of soluble Aβ and in amyloid plaque deposition [28].

Garcia-Alloza et al. carried out a study with AD-induced APPswe/PS1d9 mice. The oral administration of VE (210 mg/kg) 1 day before and 15 days after surgery was able to reduce AD progress, decrease oxidative stress, and reduce damage to the neurite structures [30].

In addition, Bostanci et al. administered α-Toc (100 mg/kg/day) for 10 days to Wistar rats previously induced to neurotoxicity and oxidative stress, after which they found that the loss of neurons was attenuated and a neuroprotective effect was observed [34].

Singh and Chauhan induced Parkinson’s-like symptoms in Wistar rats, followed by the administration of tocopherol (5 and 10 mg/kg) intraperitoneally for 40 days. Such a procedure attenuated behavioral changes, in addition to improving the expression of neurotransmitters and reducing the levels of inflammatory markers [45].

Iqbal et al. carried out a study with Swiss albino mice with PD induced by haloperidol. Oral administration of tocopherol (5, 10, 20, and 40 mg/kg) for 23 days was able to increase the levels of antioxidant enzymes and neurotransmitters and decrease the levels of inflammatory cytokines and α-synuclein mRNA expression [46].

#### 3.1.5. Neuroprotection and Neuroregeneration

VE and α-Toc have shown a neuroprotective effect in several neurodegenerative models. Supplementation with vitamin E and α-Toc has been able to reach the entire CNS, hippocampus, neurons, memory functions, behavior, learning, and cognition, thus protecting from oxidative stress, which is the main cause of neurodegenerative disorders [28,29,30,31,32,34,35,36,37,38,41,43,44].

When VE was administered to transgenic mice APPswe/PS1d9 with induced AD, a regenerative effect was observed, thereby slowing the progress of AD, reducing oxidative stress, and altering structures in neurons that resulted in neuroprotection [30].

**Figure 2:**
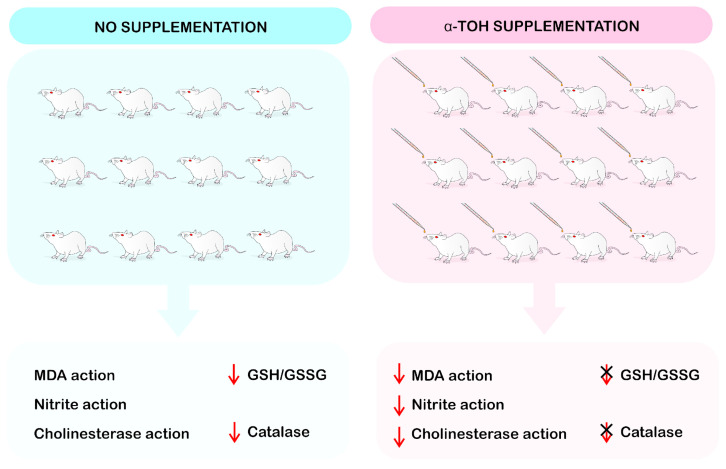
Biochemical effects of vitamin E supplementation in rats. The groups treated with α-TOH (α-tocopherol) supplementation resulted in reduced levels of malondialdehyde (MDA), nitrites, and cholinesterase, in addition to preventing the loss of glutathione (GSH) and oxidized glutathione (GSSG) when compared to non-supplemented animals, which had a decrease in GSH/GSSG and Catalase (Illustration performed by Dr. Francisco Irochima).

In another study, Wang et al. [39] used APPswe/Ps1dE9 mice with AD and supplemented them with 100 mg/kg of α-Toc for four weeks, while Liu et al. [40] used C57Bl/6J mice with induced cognitive deficit and oxidative stress and supplemented them with VE for seven days at 50 mg/kg/day. On the other hand, Rana et al. [42] induced cognitive deficit and motor impairment in Wistar rats by means of TBI, followed by supplementation with α-Toc for 28 days at 5–10 mg/kg. These authors reported that vitamin E and α-Toc supplementation was able to protect and regenerate the CNS of the animals, despite the diversity of induced neurodegenerative diseases, the doses of VE and α-Toc, and the heterogeneity in the duration of the experiments (Figure 2).

## 4. Discussion

The studies analyzed in this review evidenced that VE supplementation is able to reduce oxidative stress and lipid peroxidation as well as inhibit the brain inflammatory process in animal models of neurodegenerative diseases. Considering that there is scientific evidence that links oxidative stress to CNS inflammation with the onset and progression of neurodegenerative diseases, the results of this review point to a beneficial effect of supplementation with foods rich in α-tocopherol.

The studies included in this review showed that VE supplementation had a positive effect on oxidative stress through a reduction in lipid peroxidation and senile plaques as a result of its anti-inflammatory effect on the CNS. VE has the ability to regulate the gene expression of proteins involved in the cellular redox state and, consequently, oxidative stress. In fact, the studies demonstrate that α-Toc supplementation acts on lipid peroxidation [47]. Similarly, a previous study reported that vitamin E has fat-soluble antioxidant capacity, efficiently eliminating membrane lipid peroxidation and protecting against free radical damage while maintaining membrane integrity [18]. In this perspective, a study conducted by Takatsu and colleagues demonstrated that vitamin E improves the cognitive deficit caused by aging, not only through its neuroprotective activity but also through its antioxidant effect [48].

Another study demonstrated the potential ability of VE to counteract the effects of traumatic brain injury (TBI) on the molecular substrates underlying synaptic plasticity and cognitive function in the hippocampus in rat models. The results suggested that dietary VE supplementation may protect the brain against the effects of mild TBI on synaptic plasticity and cognition by using molecular systems associated with long-term maintenance of synaptic plasticity, such as BDNF and its downstream effectors in preserving activity, synaptic, synapsin I, CREB, and CaMKII [49]. Other studies in this review corroborate this neuroregenerative effect, which has resulted in delaying the progression of AD, increasing cell number, reducing cell damage, changing neurite structure, reducing oxidative stress, and significantly improving cognitive functions [30,39,40].

Mangialasche and colleagues reported in their study that low levels of vitamin E are found in older adults with AD and mild cognitive impairment [50]. From this perspective, the relationship between increased levels of VE and a reduction in the incidence of cognitive impairment cannot be ruled out. Based on these results, it is understood that high levels of VE (α-Toc and γ-tocopherol) might play a preventive role in reducing the risk of cognitive impairment [51]. Therefore, it is possible to infer that the elevation of plasma α-Toc may result in better global cognition and a reduction in total brain atrophy [52]. In fact, different authors found similar results, showing that VE supplementation is a potential alternative to improve the cognitive function of animals [29,31,33,35,36,37,39,42,43,44].

Furthermore, this review analyzed studies in which VE and α-Toc were able to cause a significant improvement in motor activity by reducing the Bax/Bcl-2 ratio in the prefrontal cortex, striatum, substantia nigra, and globus pallidus, in addition to restoring neurotransmitter levels. Therefore, VE and α-Toc supplementation have demonstrated a potential effect on motor changes caused by neurodegenerative diseases through a reduction in stereotyped behaviors and functional decline [29,38,42,52]. In fact, previous studies have reported that the substantia nigra is an area of the midbrain responsible for controlling motor activity, which indicates that neurodegeneration of dopaminergic neurons in this region can result in the genesis of Parkinson’s disease (PD) and other motor dysfunctions [53].

Ulatowski et al. reported that VE deficiency causes dysfunction in the cerebellum, degeneration of its neurons, premature death, and ataxia [54]. Purkinje cerebellar cells also undergo deterioration, being the main integrators of neural circuits in this region. VE-deficient TTP null mice show a high degree of atrophy of Purkinje neurons and reduced neuronal connectivity, highlighted by reduced neuronal arborization. In this perspective, Yokota et al. demonstrated that α-Toc supplementation almost completely corrected the abnormalities in a mouse model with α-TTP gene mutations that are linked to isolated LV ataxia deficiency (AVED). These authors concluded that α-tocopherol supplementation suppressed lipid peroxidation and almost completely prevented the development of neurological symptoms [55].

El-Shaer et al., using an experimental model of exposure to an electromagnetic field, evaluated the effects on the structural properties of the cerebellum and the possible neuroprotective effects of VE. The submission to magnetism caused demyelination and degeneration of axons in the molecular and granular layers, as well as a reduction in Purkinje cells and pyknotic nuclei. However, supplementation with VE enabled a reduction in neuropathological changes in all the aforementioned cerebellar areas [56].

In addition, some studies have reported that VE supplementation is able to reduce the neurodegenerative process involved in PD. This disease is characterized by the degeneration of dopaminergic neurons in the substantia pars compacta in the substantia nigra. In addition, it is associated with reduced levels of dopamine in the nigrostriatal pathway in the brain [57,58]. Although the genesis of PD is uncertain, oxidative stress is one of the main mechanisms involved in its underlying pathophysiology [57,59]. This review demonstrated that VE was able to reduce the progression of the degenerative process associated with PD through a reduction in oxidative stress. Therefore, it is believed that vitamin E causes neuronal resistance and recovery of the atrophied neurons, thus delaying their functional decline [32,45,46].

VE administration (α-Toc and Trolox) has the ability to reverse synaptic plasticity abnormalities in PINK1 -/- mice. The PINK1 haploinsufficiency precipitates mitochondrial functioning, impairing mitophagy and energy production on demand, therefore being responsible for the reduced release of synaptic vesicles at dopaminergic terminals and the subsequent breakdown of corticostriatal synaptic plasticity [60,61,62,63]. In this review, Rana and colleagues found similar results in which αToc restored altered neurotransmitter levels after TBI induction [42]. In this study, VE and α-Toc positively interfered in inhibiting or inactivating neuroinflammatory and neuroprotective substances, as well as markers of oxidative stress in the CNS, through a reduction in GSH/GSSG, catalase, and superoxide dismutase (SOD) activity, in addition to the mediators interleukin-1 beta (IL-1β), IL-6, and tumor necrosis factor-alpha (TNF-α).

The antioxidant system has other important components, such as SOD, which plays a fundamental role in protecting cells against the deteriorating and ROS scavenging effects [64,65], portraying the function of SOD as having the ability to catalyze the conversion of superoxide radicals to hydrogen peroxide and molecular oxygen, whereas CAT catalyzes the breakdown of toxic H_2_O_2_ into water and oxygen. In addition, there are other enzymes that inactivate hydrogen peroxide, a potent ROS that has the ability to convert into a stable product, such as glutathione peroxidase (GPx) [66]. Ward and colleagues [67] stated that the aging process causes different cellular changes, such as increased intracellular Ca^2+^ levels, which cause low-grade inflammation in the CNS and peripheral systems. Furthermore, they reported that low-grade inflammation causes the release of IL-1β, IL-6, and TNF-α. However, it has been reported that VE and/or LTB supplementation decreases TNF-α and IL-6 levels, reducing DNA fragmentation and MAPK signaling pathways. Thus, VE supplementation was able to inhibit oxidative damage in vivo in systemic vasculitides [68,69].

In addition, Celikoglu et al. [70] demonstrated that VE alleviated the oxidative stress induced by mercury, causing increased activities of SOD, catalase, and glutathione peroxidase. Furthermore, α-Toc supplementation resulted in a protective effect against oxidative brain damage through a reduction in lipid peroxidation and improved brain antioxidant capacity by increasing GSH levels [71,72].

Studies carried out using elderly rodents have shown that the BDNF system is affected at several levels with aging, which includes reduced transcription, synthesis, and protein processing [73]. These reductions are related to hippocampal shrinkage [74], spatial memory decline [75], and neuronal atrophy [76]. However, VE was able to promote an enhancement in cognition mediated by BDNF through its ability to regulate the production of CaMK in the hippocampus, whereas α-Toc was able to increase the molecular factors associated with memory consolidation such as BDNF, CREB, and synapsin I, all of which can be affected by oxidative stress [49,77,78].

Although the studies with animal models of neurodegenerative diseases that underwent supplementation with VE or α-Toc have shown potential in preventing and controlling the progression of these diseases, there are limitations in this review that may compromise the extrapolation of results. The diversity found in the amount of supplementation used, as well as in the administration route and in the heterogeneity of the evaluation of the effects on the CNS, are limitations that need to be pointed out. In addition, it is important to emphasize that only in vivo studies were included in this review, and even with scientifically validated evidence, there is a conviction that the results cannot yet be fully extrapolated to humans.

## 5. Conclusions

VE and α-Toc have the potential to significantly improve cognition, memory, learning, motor function, and disease progression in animal models of neurodegenerative diseases, playing a preventive role.

Therefore, the promising results shown in the in vivo studies indicate that vitamin E can reduce oxidative stress and lipid peroxidation and inhibit the brain inflammatory process, whose mechanisms are present in different neurodegenerative diseases. However, there is a need to confirm these findings in controlled clinical trials that assess the efficacy of vitamin E supplementation in humans with such CNS pathologies.

## Figures and Tables

**Table 1:** Characteristics and main findings of the studies included in this review.

Reference	Species/Strain	Gender	Control Group	Induction Method	Experimental Model	Type of Subst.	Duration	Dose	Admin. Route	Data Collection Time	Outcome Measurement	Main Findings
Sung 2003 [28]	Tg 2576 mice	M/F	Placebo	Transgenic rats	AD ^a^	Vitamin E	6 to 8 months	2 mg/g	Oral	8 months	Neuroprotection	Reduced lipid peroxidation, soluble Aβ ^b^ and amyloid plaque deposition
Conte et al., 2004 [29]	Tg 2576 mice	F	Normal chow	RCBI ^c^	AD ^a^	Vitamin E	4 weeks before lesion and 8 weeks after	2 mg/g	Injection into the left parieto-temporal region.	8 weeks	Neuroprotection	Reduced BLP (Brain Lipid Peroxidation) Alleviated the learning deficit Improved behavioral commitment
Garcia-Alloza 2006 [30]	APPswe/PS1d9 mice	*	2 received distilled water 2 received cremophor 25% in distilled water	Transgenic rats	AD ^a^	Trolox (Vitamin E)	15 days	1 day before sugery 210 mg/kg, and after for 15 days	Gavage	15 days	Neuroregeneration	Slowed the progress of AD ^a^ Significantly reduced oxidative stress Changed the structures of the neurites
Annaházi, 2007 [31]	Wistar rats	M	Operated group Nothing applied	Chronic brain hypoperfusion	Brain injury in bilateral common carotid arteries	α-tocopherol	5 days before surgery and 5 days after	100 mg/kg	Intraperitoneal	17 days	Neuroprotection	Improved the learning process Prevented loss of stained pyramidal cells in the CA1 hippocampus Preserved dendritic arborizations Attenuated microglial activation
Pasbakhsh, 2009 [32]	Sprague-Dawley rats	M	Sham group operated + Sham group treated with vehicle + sham group treated with vitamin E	6-OHDA ^d^	PD ^e^ initial model	Vitamin E	8 weeks	D-a-tocopheryl succinate (16 mg/kg, i.m, Bioglan, UK) and 0.8 mL/kg of propylene glycol	Intramuscular	2 weeks after surgery	Neuroprotection	Delayed functional decline
Tiwari et al., 2009 [33]	Wistar rats	M	Injected citrate buffer	ST’Z ^f^	AD ^a^ Cognitive deficit	α-tocopherol	21 days after lesion	100 mg/kg	Oral	21 days	Neuroprotection	Prevention of cognitive impairment Prevented a reduction in the levels of GSH ^g^ and catalase Reduced MDA ^h^, nitrite and cholinesterase activity
Bostanci, 2010 [34]	Wistar rats	M	Saline solution	Iron	Neurotoxicity Oxidative stress	α-tocopherol	10 days	100 mg/kg/day	Intraperitoneal	10 days	Neuroprotection	Attenuated the loss of neurons Decreased cell loss in the hippocampus and substantia nigra Protective effect on pyramidal cells of the hippocampus
Alzoubi, 2012 [35]	Wistar rats	M	Vehicle	Sleep deprivation	Memory deficit Learning impairment	Vitamin E	6 weeks	100 mg/kg	Oral by gavage	6 weeks	Neuroprotection	Prevented memory impairment It normalized the reduction in oxidative stress markers (GSH ^g^/GSSG ^i^), SOD ^j^ and GPx ^k^ catalase activity
Desrumaux, 2013 [36]	PLTO-KO mice	M	3 μL of vehicle	A*β* ^b^ 25–35	AD ^a^ Memory impairment	Vitamin E	10 days	800 mg/kg	Oral	10 days	Neuroprotection	Reduced short-term memory impairment Prevented PLTP-KO ^l^ compromise
Ishihara, 2013 [37]	3 Tg—AD ^a^ mice	*	Normal diet	Transgenic rats	AD ^a^ pathogenesis Cognitive deficit	α-tocopherol	4 months	1.342 mg/g and normal diet 0.076 mg/g	Oral	4 months and 4 days	Neuroprotection	Prevented cognitive impairment Attenuated the reduction in GSH ^g^ levels and the increase in GSSG ^i^ and TBARS ^m^ Decreased the levels of reactive radicals in the brain
An, 2016 [38]	Sprague Dawley rats	M	Saline solution	HAL ^n^	Orofacial dyskinesia VCM ^o^	α-tocopherol	5 weeks	20 mg/kg/day	Oral	5 weeks from the last administration	Neuroprotection	Reduced stereotypical behavior Decreased the expression of anti-apoptotic protein Bcl-2 ^p^ Increased the expression of pro-apoptotic Bax ^q^ protein Decreased Bax ^q^/Bcl-2 ^p^ ratio in prefrontal cortex, striatum, substantia nigra, and globus pallidus
Wang, 2016 [39]	APPswe/PS1dE9 mice	M	Saline solution	Transgenic rats	AD ^a^	α-tocopherol	4 weeks	100 mg/kg	Oral gavage	4 weeks	Neuroprotection Neuroregeneration	Improved memory impairment Improved cognitive dysfunction Counteracted oxidative stress Decreased the levels of Aβ ^b^ oligomer
Liu, 2019 [40]	C57BL/6J mice	M	0 mg kg –1 day –1 of PM 2.5 ^r^	PM 2.5 ^r^	Cognitive deficit Oxidative stress	Vitamin E	7 days	50 mg/kg^–1^ day^–1^	Intragastric	7 days	Neuroprotection Neuroregeneration	Improved cognitive function Reduced cellular damage Increased the number of cells Decreased the expression of Aβ ^b^ 1–42 Reduced oxidative stress
Jahanshahi, 2020 [41]	Wistar rats	M	No medication	Scopolamin	AD ^a^	Vitamin E	14 days after induction	25, 50, and 100 mg/kg/day	Intraperitoneal	16 days	Neuroprotection	Amyloid plaque reduction Prevented an increase in neurofibrillary tangles in hippocampal subregions
Rana et al., 2020 [42]	Wistar rats	M	Vehicle	TBI ^s^	Cognitive impairment Motor damage	α-tocopherol	28 days after induction	5 mg/kg, po. 10 mg/kg, po.	Weight drop model	28 days	Neuroprotection Neuromodulation	Attenuated locomotor performance Reduced cognitive impairment Reduced neuroinflammatory markers Restored neurotransmitter levels Balanced oxidative stress
Nesari, 2021 [43]	Wistar rats	M	DMSO ^t^ + Saline solution	Lactacystin	Proteasome inhibition Oxidative stress Memory impairment	α-tocopherol	5 days before induction	60 and 200 mg/kg, i.p.	Bilateral hippocampal injection	7 days	Neuroprotection	High doses of α-tocopherol exhibited remarkable mitochondrial protection Improved memory impairment Increased the levels of glutathione
Shahidi, 2021 [44]	Wistar rats	M	Saline solution + Non operated	Aβ ^b^ 25–35	AD ^a^	Vitamin E	10 days	200 mg/kg	Oral by gavage	2 weeks 10 days	Neuroprotection	Improved passive avoidance of memory impairment The amplitude of the PS ^u^ was increased Alleviated LTP ^v^ deficiency Reverted the increase in Bcl-2 ^p^ and Bax ^q^ ratio in the hippocampus
Singh, 2022 [45]	Wistar rats	M	Ropinirol	Rotenona	Sintomas semelhantes a PD ^e^	Tocopherol	40 days	5 and 10 mg/kg	Intraperitoneal	41 days	Neuroprotection and neuroinflammation	Attenuated behavioral changes Enhanced the expression of neurotransmitters Reduced the levels of inflammatory markers
Iqbal, 2022 [46]	Albino swiss mice	M	CMC ^w^	HAL ^n^	PD ^e^	Tocopherol	23 days	5, 10, 20 and 40 mg/kg	Oral route	23 days	Neuroprotection	Increased the levels of antioxidant enzymes and neurotransmitters Decreased the levels of inflammatory cytokines and the expression of α-synuclein mRNA

* = not mentioned; ^a^ AD = Alzheimer’s disease; ^b^ Aβ = β-amyloid; ^c^ RCBI = repetitive concussive brain injury; ^d^ 6-OHDA = 6-hydroxydopamin; ^e^ PD = Parkinson’s disease; ^f^ STZ = streptozotocin; ^g^ GSH = glutathione; ^h^ MDA = malondialdehyde; ^i^ GSSG = oxidized glutathione; ^j^ SOD = superoxide dismutase; ^k^ GPx = glutathione peroxidase; ^l^ PLTP-KO = plasma phospholipid transfer protein-deficient; ^m^ TBARS = thiobarbituric acid-reactive substance; ^n^ HAL = haloperidol; ^o^ VCM = vacuum chewing movements; ^p^ BCL-2 = B-cell lymphoma 2; ^q^ BAX= B-cell lymphoma 2 associated protein X; ^r^ PM 2.5 = ambient fine particulate matter (aerodynamic diameter < 2.5 um, PM 2.5); ^s^ TBI = traumatic brain injury; ^t^ DMSO = dimethylsulfoxide; ^u^ PS = population increase; ^v^ LTP = long-term potentiation; ^w^ CMC = carboxymethylcellulose.

## Supplementary Materials

The following supporting information can be downloaded at: https://www.mdpi.com/article/10.3390/ijms241311191/s1.
