# Supplementary material for: Vitamin E and Its Molecular Effects in Experimental Models of Neurodegenerative Diseases
Source: Int J Mol Sci. 2023 Jul 7;24(13):11191. doi: 10.3390/ijms241311191 (PMC10342514; doi:10.3390/ijms241311191)
Supplement: Supplementary file 1 [file ijms-24-11191-s001.zip › ijms-2426546-supplementary.pdf]

Table S1 - Final search strategy performed on Medline/PubMed

Search	Search strategy	Found items
#3	#1 AND #2	365
#2	**"alpha-Tocopherol"[Mesh]** OR (alpha Tocopherol) OR (3,4-Dihydro-2,5,7,8-tetramethyl-2-(4,8,12-trimethyltridecyl)-2H-1-benzopyran-6-ol) OR (d-alpha Tocopherol) OR (Tocopherol, d-alpha) OR (d alpha Tocopherol) OR (R,R,R-alpha-Tocopherol) OR (alpha-Tocopherol Succinate) OR (alpha Tocopherol Succinate) OR (Tocopherol Succinate) OR (alpha-Tocopherol Hemisuccinate) OR (alpha Tocopherol Hemisuccinate) OR (Vitamin E Succinate) OR (d-alpha-Tocopheryl Acetate) OR (d alpha Tocopheryl Acetate) OR (alpha-Tocopherol Acetate) OR (alpha Tocopherol Acetate) OR (Tocopheryl Acetate) OR (Tocopherol Acetate) OR (Acetate, Tocopherol) OR (alpha-Tocopheryl Calcium Succinate) OR (alpha Tocopheryl Calcium Succinate)	
#1	**"Neurodegenerative Diseases"[Mesh]** OR (Neurodegenerative Disease) OR (Degenerative Diseases, Neurologic) OR (Neurologic Degenerative Disease) OR (Degenerative Neurologic Diseases) OR (Degenerative Neurologic Disease) OR (Neurologic Disease, Degenerative) OR (Neurologic Diseases, Degenerative) OR (Nervous System Degenerative Diseases) OR (Neurodegenerative Disorders) OR (Neurodegenerative Disorder) OR (Neurologic Degenerative Conditions) OR (Degenerative Condition, Neurologic) OR (Degenerative Conditions, Neurologic) OR (Neurologic Degenerative Condition) OR (Neurologic Degenerative Diseases) OR (Degenerative Diseases, Nervous System) OR (Degenerative Neurologic Disorders) OR (Degenerative Neurologic Disorder) OR (Neurologic Disorder, Degenerative) OR (Neurologic Disorders, Degenerative) OR (Degenerative Diseases, Spinal Cord) OR (Degenerative Diseases, Central Nervous System)	
